# Association of Lipoprotein A rs10455872 Polymorphism with Childhood Obesity and Obesity-Related Outcomes

**DOI:** 10.3390/diagnostics15141809

**Published:** 2025-07-18

**Authors:** Ayşen Haksayar, Mustafa Metin Donma, Bahadır Batar, Buse Tepe, Birol Topçu, Orkide Donma

**Affiliations:** 1 Department of Child Health and Diseases, Faculty of Medicine, Tekirdağ Namık Kemal University, 59030 Tekirdağ, Türkiye; mmdonma@nku.edu.tr; 2 Department of Medical Biology, Faculty of Medicine, Tekirdağ Namık Kemal University, 59030 Tekirdağ, Türkiye; bbatar@nku.edu.tr; 3 Department of Medical Genetics, Faculty of Medicine, Tekirdağ Namık Kemal University, 59030 Tekirdağ, Türkiye; busettepe@gmail.com; 4 Department of Biostatistics, Faculty of Medicine, Tekirdağ Namık Kemal University, 59030 Tekirdağ, Türkiye; btopcu@nku.edu.tr; 5 Department of Medical Biochemistry, Faculty of Medicine, Istanbul University—Cerrahpasa, 34320 Istanbul, Türkiye; donmaohm@iuc.edu.tr

**Keywords:** childhood obesity, genotypes, lipoprotein(a), polymorphism, real-time PCR

## Abstract

**Background/Objectives:** Obesity is associated with cardiovascular disease worldwide. An increased lipoprotein A (LpA) level is an independent risk factor for cardiovascular disease in children. Genetic polymorphisms of the *LPA* gene may play an important role in susceptibility to obesity. The aim of this study was to investigate the association of *LPA* rs10455872 polymorphism with the risk and clinical phenotypes of childhood obesity. **Methods:** This study included 103 children with obesity and 77 healthy controls. Genotyping of the *LPA* rs10455872 polymorphism was performed using real-time PCR. **Results:** The genotype distributions of the *LPA* rs10455872 polymorphism did not differ significantly between children with obesity and healthy children (*p* = 0.563). A marked difference in insulin levels was observed between children with obesity carrying the AG (16.90 IU/mL) and AA (25.57 IU/mL) genotypes. A marked difference was also observed in CRP levels between children with obesity with the AG (2.31 mg/L) and AA (4.25 mg/L) genotypes. After correcting for multiple comparisons using the false discovery rate (FDR), significant differences were found between AG and AA genotypes in vitamin B12 (adjusted *p* = 0.024). Serum iron showed a borderline association (adjusted *p* = 0.072). A statistically significant correlation was found between the metabolic syndrome index and body fat ratio among children with obesity with the AA genotype (*p* = 0.028). **Conclusions:** Although limited by the small number of children with obesity with the AG genotype, some differences were noted between the AG and AA genotypes. These exploratory findings require further investigation in adequately powered studies. In children with obesity with the AA genotype, the metabolic syndrome index increases as the body fat ratio increases.

## 1. Introduction

Obesity is a complex chronic disease involving abnormal and excessive adiposity [1]. Obesity is a major risk factor for cardiovascular disease worldwide. An increase in an individual’s body mass index (BMI) increases the risk of cardiovascular disease. Obesity is diagnosed by using anthropometric measurements to estimate body fat. BMI is a simple method used in epidemiological studies to determine body fat percentage and is calculated by dividing the individual’s body weight in kilograms by the square of their height in meters [2].

Childhood obesity is emerging as a major public health concern [3,4,5]. The number of overweight children under the age of 5 with obesity was estimated at 38.2 million in 2019 [3]. In the European region, Türkiye ranks first as the country with the highest prevalence of overweight and obesity. The rate of obesity for both sexes in Turkey is 32.1%. In the adult population, 66.8% of individuals were reported to be overweight, including obesity, at a proportion of 69.3% for women and 64% for men [6]. It is predicted that more than 50% of adults in the United States will be clinically obese by 2030, and the prevalence of overweight in children will nearly double by 2030 [7].

Genetic alterations may increase adolescents’ susceptibility to weight gain. The interaction of genetic and environmental factors significantly affects the development of obesity. Lipoprotein a (Lpa) is a complex molecule found in human plasma, consisting of apolipoprotein A (APO A), a high-molecular-weight glycoprotein with the LDL molecule, and its plasminogen (PLG) homolog. It remains unclear whether BMI affects Lpa plasma levels in children.

The LPA gene evolved from the PLG gene. PLG contains five different K domains (KI-V) and protease domains. Kringle V and the protease domain are conserved. The protease domain is inactivated by several mutations [8]. LDL is the major cholesterol transporter in human plasma. Lpa is one of the two main ligands for the LDL receptor, which facilitates the uptake of LDL by the parenchymal cells of the liver [9].

The lipoprotein locus encompasses a region of >130 kb on chromosome 6. LPA rs10455872 (intron A/G) single-nucleotide polymorphisms are associated with elevated plasma Lpa levels. Lpa was found to be a risk factor for cardiovascular disease [10]: genetic and clinical studies have demonstrated that Lpa is related to coronary heart disease (CHD) [11], and elevated Lpa levels are independent and causal risk factors for atherosclerotic cardiovascular disease. Lpa levels can maintain a very stable course in an individual for many years. Therefore, unlike many other biomarkers, the effect of Lpa on cardiovascular disease risk can be evaluated using a single measurement [12]. In people with obesity, Lpa is altered.

The aim of this cross-sectional case–control study was to investigate the effect of the LPA rs10455872 polymorphism on the risk and clinical phenotypes of childhood obesity.

## 2. Materials and Methods

### 2.1. Study Participants

This study included 103 children with obesity and without acute or chronic illnesses and 77 healthy children with a normal BMI. The participants were aged 6–18 years and had presented at the Tekirdağ Namık Kemal University, Medical Faculty Hospital, Department of Pediatrics, Pediatric Health and Diseases Polyclinic between 1 December 2021 and 31 October 2022; 53.9% were (*n* = 97) female, and 46.1% (*n* = 83) were male.

Informed consent was obtained from all participants or the families of the children, particularly their parents, before the study commenced. After conducting a detailed pediatric physical examination and informing the parents and children about their involvement with this study, the parents signed a consent form, and a verbal agreement was made with the children who agreed to participate in the study. Children who were living with obesity according to anthropometric measurements and children with a normal body weight were included. The study was approved by the Tekirdağ Namık Kemal University Faculty of Medicine Non-Invasive Clinical Research Ethics Committee (GOKAEK 30.11.2021/2021.182.06.12).

### 2.2. Anthropometric Measurements

In the anthropometric evaluations, body weight, height, head circumference, neck circumference, waist circumference, hip circumference, and body fat ratio were measured. All measurements were performed by the same person. Body Mass Index-for-age z-scores (zBMI) were calculated using the WHO AnthroPlus software version 1.0.4, based on the 2007 WHO growth reference for children and adolescents aged 5 to 19 years. These standardized scores were used to quantify the degree of obesity among participants.

### 2.3. Index Measurements

Index measurements were calculated using the formulas below.

BMI = Body Mass Index Body weight (kg)/Height (m^2^); HOMA-IR = Homeostatic Model Assessment Insulin Resistance [Fasting blood glucose (mg/dL) × fasting insulin (µIU/mL)/22; 5 × 0.0555]; DONMA I = Diagnostic Obesity Nomination Model Assessment Index-I [body weight (kg) × 100/height (cm)]; DONMA II = Diagnostic Obesity Nomination Model Assessment Index-II [total body fat weight (kg) × 100/height (cm)]; SII index = Systemic Immuno-Inflammation Index: Platelet count × Neutrophil count/Lymphocyte count; and Metabolic Syndrome Index = [(Insulin/Fasting blood glucose)/(High-density lipoprotein/Triglyceride)].

### 2.4. Blood Sampling and DNA Extraction

Two-milliliter venous blood samples were collected from the children with obesity and the healthy controls into EDTA tubes. Genomic DNA was extracted from peripheral blood mononuclear cells (PBMCs) using a DNA extraction kit (High Pure PCR Template Preparation Kit, Roche, Basel, Switzerland) according to the manufacturer’s instructions. Genomic DNA was analyzed for purity and concentration using a NanoDrop spectrophotometer (AllSheng 100, Hangzhou, China).

### 2.5. Genotyping

Genotype distributions of the lipoprotein A single-nucleotide polymorphism (rs10455872, A > G, intron 25) were determined via real-time PCR and melting curve analysis using the LightCycler 2.0 instrument (Roche Diagnostics). The real-time PCR was performed using the LightSNiP rs10455872 assay (TIBMolBiol, Berlin, Germany) and FastStart DNA Master HybProbe (Roche Diagnostics), according to the manufacturer’s instructions.

### 2.6. Statistical Analysis

Statistical analysis was performed using SPSS 28.0 (SPSS Inc., Chicago, IL, USA), a statistical software package. In the analysis of data, descriptive statistics such as the mean, standard deviation, frequency, percentage, and tables and graphs were used. Independent samples *t*-tests were used for two-group comparisons of continuous variables. To control for the risk of false positives due to multiple testing, the FDR correction was applied to all *p*-values. Spearman’s correlation coefficient (*p*) was used to evaluate the relationships between zBMI and DONMA I and II indices separately in children with obesity and controls. Pearson correlation analysis was used to determine the relationship between the metabolic syndrome index and body fat ratio. Pearson’s chi-square (χ^2^) or Fisher’s exact test was used to determine allele or genotype frequencies and test for the deviation of genotype distribution from the Hardy–Weinberg equilibrium (HWE) and relationships between demographic and clinical characteristics. Receiver operating characteristic (ROC) curve analysis was used to evaluate the ability of the metabolic syndrome and SII indexes to predict childhood obesity. The results are presented as the area under the curve (AUC). Adjusted *p*-values less than 0.05 were considered statistically significant. Both nominal and FDR-adjusted *p*-values were reported.

The primary aim of the study was to compare the distribution of rs10455872 genotypes between children with obesity and healthy controls. However, due to the limited number of individuals with the AG genotype in our cohort, additional analyses examining associations between genotypes and biochemical analysis (e.g., B12, TSH, serum iron) within the group were considered exploratory. These analyses were performed to identify potential trends and to generate hypotheses for future studies with larger sample sizes.

## 3. Results

Normal BMI percentiles between 15 and 85 were used for the healthy controls, and BMI values at the 95th percentile and above were used for children with obesity. The mean zBMI of the children with obesity (*n* = 103) was 2.81 ± 1.05, with values ranging from 1.63 to 9.35. According to WHO criteria, all participants exceeded the threshold for obesity (zBMI > +2 SD), with some presenting with extreme obesity (zBMI > +3 SD). In the control group (*n* = 77), the mean zBMI was −0.08 to ±0.69, ranging from −1.03 to +1.10, consistent with a normal weight distribution. The index values and anthropometric characteristics of the children with obesity and the healthy controls are shown in Table 1. The differences in BMI, head/neck circumference ratio, waist/hip circumference ratio, DONMA index I and II, body fat ratio, and basal metabolic rate (BMR) between the healthy controls and children with obesity were found to be significant (*p* < 0.01).

**Figure 1:**
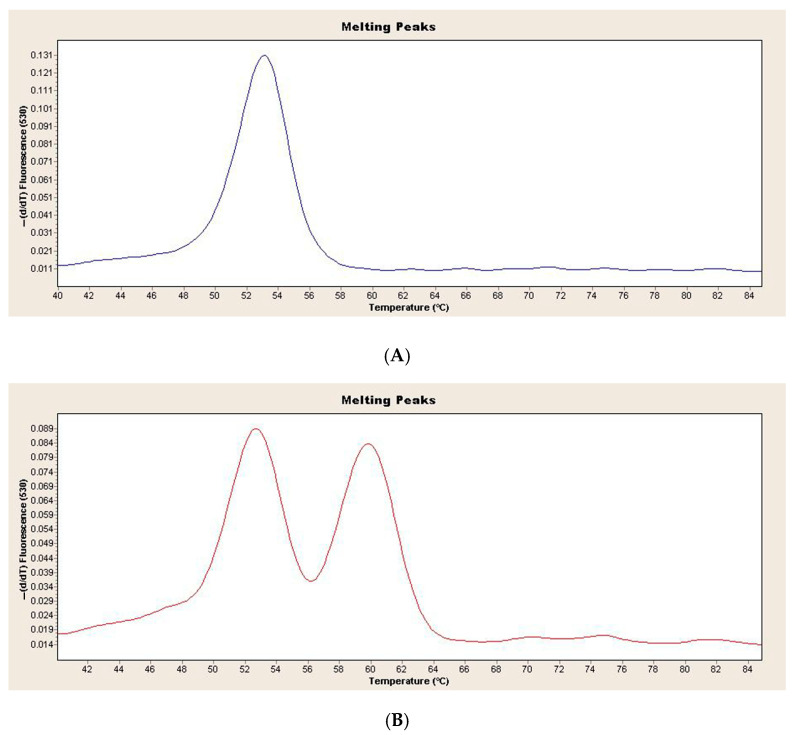
(**A**) Melting curve analysis of LPA rs10455872 wild-type genotype in children with obesity and healthy controls (**B**) Melting curve analysis of LPA rs10455872 heterozygous genotype in children with obesity and healthy controls.

Figure 1 shows the melting curve analysis of the wild-type and heterozygous genotypes in the children with obesity and healthy controls (Figure 1).

In the children with obesity, 97 wild-type AA genotypes and 6 AG genotypes were found. In the healthy controls, 73 wild-type AA genotypes and 4 AG genotypes were found. There were no significant differences in genotype distribution between the children with obesity and the healthy controls (*p* > 0.563). The distribution of *LPA* genotypes in the children with obesity and the healthy controls was not significantly different from that of the HWE (*p* = 1.00, *p* = 1.00, respectively). Table 2 shows the genotype distribution of demographic and clinical characteristics in the children with obesity. No significant difference in demographic and clinical phenotypes was found in the children with obesity according to the AA and AG genotypes (*p* > 0.05).

Of the participating children with obesity, 48 (46.6%) were female and 55 (53.4%) were male. There was no statistically significant difference in the distribution of AG and AA genotypes between male and female children with obesity (*p* = 0.594). In the group of healthy children, 49 (63.6%) were female and 28 (36.4%) were male. No statistically significant difference was observed in the distribution of AG and AA genotypes between male and female healthy controls (*p* = 0.134).

Furthermore, biochemical and hormonal parameters were compared between AG and AA genotypes in the children with obesity. After applying the FDR correction for multiple comparisons, a statistically significant difference was found in vitamin B12 (adjusted *p* = 0.024). Notably, serum iron showed a trend toward statistical significance (adjusted *p* = 0.072), suggesting a potential borderline association. Although serum iron binding capacity exhibited nominal significance (*p* = 0.023), this difference did not remain significant after FDR correction (adjusted *p* = 0.184). No statistically significant differences were observed for other parameters (Table 3). Insulin levels are considered a serious risk factor for obesity. In this study, we found that insulin levels were significantly higher in children with obesity (25.06 IU/mL) compared to healthy controls (14.25 IU/mL) (*p* < 0.001). Based on genotype distribution, a marked difference in insulin levels was observed between children with obesity carrying the AG (16.90 IU/mL) and AA (25.57 IU/mL) genotypes. The CRP levels can also be considered a risk factor for obesity. In the study, we found that CRP levels were significantly higher in the children with obesity (4.14 mg/L) compared to the healthy controls (1.99 mg/L) (*p* = 0.008). When examined according to genotype distribution, a marked difference was also observed in CRP levels between children with obesity with the AG (2.31 mg/L) and AA (4.25 mg/L) genotypes. However, given the small sample size in the AG group (*n* = 6), these analyses should be considered exploratory, and the findings interpreted with caution and regarded as hypothesis-generating.

Spearman correlation analyses were conducted separately for the children with obesity and healthy controls to examine the relationship between zBMI and DONMA indices. In children with obesity, a weak but statistically significant positive correlation was found between zBMI and DONMA I index (*p* = 0.296, *p* = 0.002). Additionally, a moderate and highly significant positive correlation was observed between zBMI and DONMA II index (*p* = 0.504, *p* < 0.001). In healthy controls, a moderate positive correlation was found between zBMI and DONMA I index (*p* = 0.433, *p* < 0.001), while a strong and statistically significant positive correlation was identified between zBMI and DONMA II index (*p* = 0.665, *p* < 0.001).

**Figure 2:**
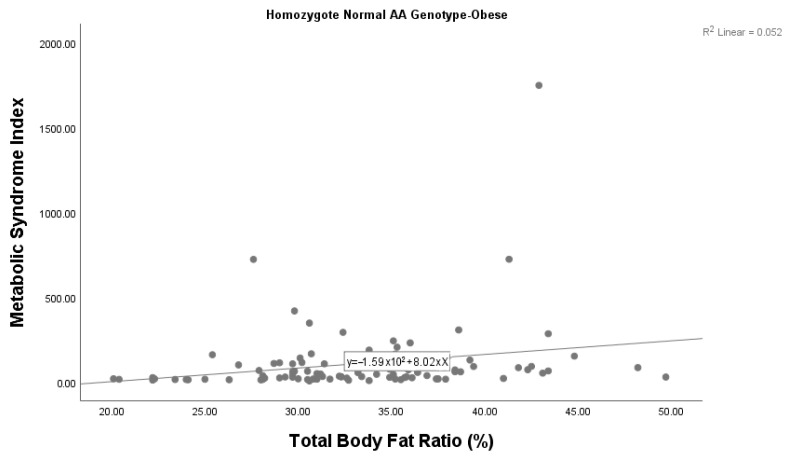
Correlation between the metabolic syndrome index and body fat ratio among children with obesity with the wild-type AA genotype.

A statistically significant correlation was also found between the metabolic syndrome index and body fat ratio among children with obesity with the AA genotype (*p* = 0.028). We found that the metabolic syndrome index increased as the body fat ratio increased in children with obesity with the AA genotype (Figure 2). There was no significant correlation between the metabolic syndrome index and body fat ratio among children with obesity who had the AG genotype (*p* = 0.606). Given the small sample size (*n* = 6), this subgroup analysis should be considered exploratory, and the findings interpreted with caution.

**Figure 3:**
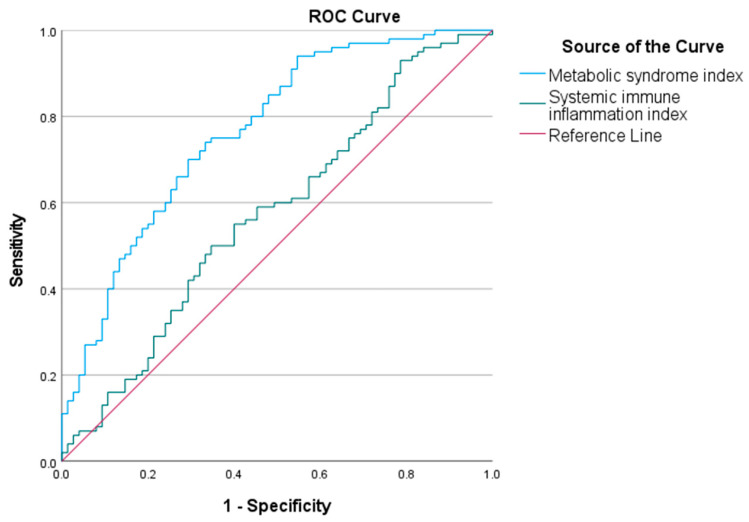
Receiver operating characteristic (ROC) curves of metabolic syndrome index and systemic immune inflammation index (SII) to predict childhood obesity. ROC analysis shows that the metabolic syndrome index (AUC = 0.767) and the SII (AUC = 0.573) have predictive value for childhood obesity. The blue curve represents the metabolic syndrome index, and the green curve represents SII.

The area under the curve (AUC) for the metabolic syndrome index in predicting childhood obesity was 0.767 (95% CI 0.70–0.84). The optimal cut-off value was 20.18. The AUC for the SII in predicting childhood obesity was 0.573 (95% CI 0.49–0.66). The optimal cut-off was 463.78. The ROC curves are shown in Figure 3.

## 4. Discussion

Factors causing obesity in children can be affected by genetic features [13]. APO A is one of the most polymorphic genes in the human genome [14]. rs10455872 is a widely studied common *LPA* single-nucleotide polymorphism (SNP). It is significantly associated with Lpa levels in Caucasians [15]. Lpa levels are highly heritable, with over 50% of all variations caused by polymorphisms in the LP(a) locus [16,17]. A genome-wide association study (GWAS) showed an association between LPA locus variations and elevated levels of Lpa [18]. LPA locus was found to be associated with the Lp(a) concentration with genome-wide significance. Various SNPs in the LPA locus have been reported to be associated with LPA mRNA expression levels in the liver and serum LP(a) levels [19,20]. rs10455872 in the *LPA* gene is a major genetic determinant of plasma Lp(a) levels, accounting for approximately 28% of its variation. Mendelian randomization studies utilizing rs10455872 (and larger kringle IV type 2 copy number variant) have consistently demonstrated a causal link between elevated Lp(a) and cardiovascular outcomes such as coronary artery disease, aortic valve stenosis, and peripheral artery disease [21]. However, causal associations between Lp(a) variants and obesity are not yet supported by Mendelian randomization data, suggesting that obesity-related mechanisms may differ in pathway specificity or effect magnitude. Plasma Lp(a) concentrations vary significantly among different ethnic groups, with individuals of African descent often exhibiting twice as high levels as Caucasians, Hispanics, and many Asian populations, while intermediate levels are suggested for South Asians. The determined interethnic difference could also be due to the allele distribution of apo(a) [22]. Furthermore, allele frequencies of rs10455872 vary by ancestry, affecting both expression and disease associations [23]. Tissue expression cluster (RNA) showed that the LPA gene is highly expressed in the liver, with minimal expression in other tissues [24]. Inflammatory cytokines such as interleukin-6 (IL-6) enhance LPA gene transcription in human HepG2 cells [25]. Although high Lpa levels are not a feature of obesity, studies have found that Lpa levels are inversely related to insulin resistance and levels [26,27]. A recent study examined Lpa levels in individuals with obesity after diet-induced weight loss, reporting an increase of 27% [28]. Some studies have shown that genetic factors and single-gene disorders may affect obesity [29]. Some loci have been reported to be more specific for obesity or morbid obesity in the childhood age group [30]. Obesity is one of the most important symptoms of Prader–Willi syndrome, which is a rare, complex genetic disease thought to occur as a result of an abnormality in the hypothalamic satiety center and growth hormone deficiency [31].

In studies conducted in adults, SNPs in the genes *FTO*, *MC4R*, *TMEM18*, *TNNI3K*, *SEC16B*, *GNPDA2*, and *POMC* are strongly associated with obesity risk [32]. In our study, no significant relationship was found when the genotype distribution of the *LPA* rs10455872 polymorphism was compared between the children with obesity and the healthy controls. No homozygous variant GG genotype was detected in the children with obesity or the healthy controls. When evaluated in terms of childhood obesity, the distribution of the *LPA* rs10455872 gene polymorphism was found to be equal to the distribution observed in the population.

The head/neck ratio and DONMA I and II indexes are measurements used in the evaluation of obesity. The DONMA II index is more valuable in the measurement of obesity than the DONMA I index [33]. Similarly, in our study, DONMA I and II values in children with obesity were found to be statistically significantly higher than in the healthy controls.

In obesity, which is associated with low-grade inflammation, the SII index is also used as a marker of inflammation [34]. In our study, although there was no statistically significant difference in the SII index between the children with obesity and the healthy controls, the mean values were higher for the individuals with obesity than for the healthy controls, demonstrating the relationship between obesity and inflammation.

In children with obesity, children with the AG genotype were compared to those with the wild-type genotype in terms of biochemical and hormonal parameters. A statistically significant difference was observed in vitamin B12 levels (adjusted *p* = 0.024), highlighting its potential relevance in the studied context. Additionally, serum iron levels demonstrated a trend toward significance (adjusted *p* = 0.072), indicating a possible borderline association. In these exploratory analyses, differences in values observed in the AG genotype subgroup may point to a potential association with obesity-related parameters; however, due to the limited sample size, these findings should be interpreted with caution and confirmed in future studies.

Serum iron and ferritin are both used to evaluate iron status, but they reflect different aspects. Serum iron measures the amount of iron bound to transferrin in the blood. Serum ferritin is an intracellular protein that stores iron; serum levels reflect total body iron stores. Donma O et al. found that RDW was significantly higher in children with obesity than in a normal-weight group [35]. However, in another study, RDW measurements did not show a statistically significant difference between children with obesity and a control group (*p* > 0.05) [36].

In the current study, significant positive correlations were observed between zBMI and both DONMA I and DONMA II indices in children with obesity and controls. Interestingly, the strength of these associations was stronger in the control group, particularly for DONMA II, compared to the obesity group. These findings suggest that increases in zBMI may be accompanied by elevations in DONMA markers, regardless of obesity status. The stronger correlations observed in the control group might indicate that DONMA indices are sensitive to early metabolic or inflammatory changes. However, it is important to interpret these results with caution. The cross-sectional design of the study does not allow for causal inferences.

A significant difference was found between the metabolic syndrome index and body fat ratio among children with obesity with the wild-type genotype. It was determined that the metabolic syndrome index increased as the body fat ratio increased in children with obesity with the wild-type genotype. While differences between AG and AA genotypes were observed, subgroup analyses involving the AG genotype (*n* = 6) should be interpreted with caution due to the small sample size and exploratory nature of the findings. Since this study is limited by the small number of samples included, these data should be confirmed by studies conducted in larger case–control groups. The findings derived from a limited number of subgroup studies may not be sufficiently reliable and thus should be interpreted with caution. A small sample size and statistical uncertainty of the study limit the statistical power. A key limitation of our study is the absence of data on pubertal status and lifestyle factors such as physical activity and dietary habits, which are known to influence obesity-related parameters. Additionally, the use of non-validated metrics such as DONMA I and DONMA II indices constitutes another important limitation. These indices have not yet been formally validated in the studied population. Future studies should incorporate these variables and focus on these measures to improve internal validity and generalizability.

In conclusion, the genotype distribution of the Lp(a) rs10455872 gene polymorphism was found to be similar in children with obesity and healthy children. In children with obesity with the wild-type genotype, a significant difference was observed between the metabolic syndrome index and body fat ratio. However, no significant difference was found for these parameters in those with the AG genotype. This exploratory analysis also identified significant differences in vitamin B12 levels between AG and AA genotypes in children with obesity. These findings should be interpreted with caution due to the limited sample size and warrant further investigation in larger, well-powered studies.

## Figures and Tables

**Table 1:** Indexes and anthropometric characteristics of healthy controls and children with obesity.

Parameters	Healthy Controls *n* = 77	Children with Obesity *n* = 103	*p*
	X ∓ ss	X ∓ ss	
BMI * (kg/m^2^)	17.39 ∓ 2.33	27.27 ∓ 5.31	<0.001
zBMI	−0.08 ∓ 0.69	2.81 ∓ 1.05	<0.001
Head/neck circumference (cm)	1.82 ∓ 0.12	1.62 ∓ 0.13	<0.001
Waist/hip circumference (cm)	0.83 ∓ 0.05	0.89 ∓ 0.07	<0.001
DONMA I **	25.76 ∓ 6.35	41.59 ∓ 11.31	<0.001
DONMA II ***	5.07 ∓ 1.98	14.05 ∓ 6.12	<0.001
HOMA-IR (mIU-mmol/L^2^) ****	3.29 ∓ 3.47	5.76 ∓ 5.76	0.001
SII (×10^9^/L) *****	454.10 ∓ 209.80	497.41 ∓ 212.05	0.177
Age	131.51 ∓ 36.22	134.51 ∓ 35.09	0.577
Body fat ratio (%)	19.37 ∓ 4.34	32.72 ∓ 6.3	<0.001
BMR ****** (kcal)	1262.53 ∓ 255.05	1615.52 ∓ 334.86	<0.001

The patient group consisted of 103 individuals, and the control group comprised 77 individuals. * BMI: Body Mass Index; ** DONMA I: Diagnostic Obesity Nomination Model Assessment Index-I, [body weight (kg) × 100/ height (cm)]; *** DONMA II: Diagnostic Obesity Nomination Model Assessment Index-II, [total body fat weight (kg) × 100/height (cm)]; **** HOMA-IR: [Fasting glucose (mg/dL) × fasting insulin (mU/L)/405]; ***** SII: Systemic immune-inflammation index: Platelet counts × Absolute Neutrophil counts/ Absolute Lymphocyte counts (×10^9^/L); ****** BMR: Basal Metabolic Rate.

**Table 2:** Genotype distribution of demographic and clinical characteristics in children with obesity.

Parameters	Children with Obesity AA *n* = 97	Children with Obesity AG *n* = 6	*p*
	X ∓ ss	X ∓ ss	
BMI * (kg/m^2^)	27.41 ∓ 5.27	25.01 ∓ 5.91	0.285
Head/neck circumference (cm)	1.62 ∓ 0.13	1.67 ∓ 0.14	0.347
Waist/hip circumference (cm)	0.89 ∓ 0.07	0.88 ∓ 0.08	0.679
DONMA I **	41.77 ∓ 11.13	38.64 ∓ 14.75	0.513
DONMA II ***	14.17 ∓ 6.04	12.16 ∓ 7.72	0.438
HOMA-IR (mIU-mmol/L^2^) ****	58.90 ∓ 58.97	38.14 ∓ 21.50	0.395
SII (×10^9^/L) *****	496.31 ∓ 216.18	515.11 ∓ 140.12	0.834
Age	134.26 ∓ 34.78	138.50 ∓ 43.20	0.776
Body fat ratio (%)	32.91 ∓ 6.14	29.65 ∓ 8.51	0.220
BMR ****** (kcal)	1617.46 ∓ 326.65	1584.16 ∓ 487.36	0.814

The patient group consisted of 103 individuals, including 97 with the AA genotype and 6 with the AG genotype. Exploratory analyses with small sample size (*n* = 6) are indicated and results should be interpreted cautiously. * BMI: Body Mass Index; ** DONMA I: Diagnostic Obesity Nomination Model Assessment Index-I; [body weight (kg) ×100/ height (cm)]; *** DONMA II: Diagnostic Obesity Nomination Model Assessment Index-II; [total body fat weight (kg) × 100/height (cm)]; **** HOMA-IR: [Fasting glucose (mg/dL) × fasting insulin (mU/L)/405]; ***** SII: Systemic immune-inflammation index: Platelet counts × Absolute Neutrophil counts/Absolute Lymphocyte counts (×10^9^/L); ****** BMR: Basal Metabolic Rate.

**Table 3:** Biochemical and hormonal parameters in children with obesity regarding genotype distribution.

Parameters	Childhood Obesity AA *n* = 97	Childhood Obesity AG *n* = 6	Nominal *p*	FDR
	X ∓ ss	X ∓ ss		Adjusted-*p*
Fasting blood sugar (mg/dL)	91.02 ∓ 6.72	90.16 ∓ 5.56	0.761	0.950
Insulin (IU/mL)	25.57 ∓ 23.76	16.90 ∓ 9.18	0.378	0.829
HbA1c (%)	5.54 ∓ 0.39	5.28 ∓ 0.27	0.116	0.464
Total cholesterol (mg/dL)	155.19 ∓ 29.27	153.13 ∓ 29.72	0.868	0.950
HDL (mg/dL)	46.23 ∓ 10.57	43.50 ∓ 5.35	0.533	0.884
Triglyceride (mg/dL)	114.27 ∓ 62.45	130.53 ∓ 63.77	0.538	0.884
LDL (mg/dL)	85.97 ∓ 26.69	88.96 ∓ 38.84	0.796	0.950
AST (IU/L)	22.40 ∓ 9.23	22.03 ∓ 5.40	0.924	0.950
ALT (IU/L)	22.77 ∓ 16.86	18.83 ∓ 5.91	0.571	0.884
CRP (mg/L)	4.25 ∓ 5.85	2.31 ∓ 2.59	0.425	0.850
Zinc (µg/dL)	100.19 ∓ 29.43	96.32 ∓ 14.83	0.795	0.950
TSH (mIU/L)	2.59 ∓ 1.14	2.12 ∓ 0.74	0.319	0.768
Vitamin B12 (pg/mL)	390.80 ∓ 148.10	624.31 ∓ 200.54	<0.001	0.024
Serum iron (µg/dL)	67.65 ∓ 27.92	101.91 ∓ 45.55	0.006	0.072
Serum iron binding capacity (µg/dL)	322.27 ∓ 57.61	266.21 ∓ 62.47	0.023	0.184
Calcium (mg/dL)	9.71 ∓ 0.38	9.58 ∓ 0.21	0.432	0.850
Phosphorus (mg/dL)	4.66 ∓ 0.63	4.42 ∓ 0.44	0.373	0.829
Magnesium (mg/dL)	2.06 ∓ 0.13	2.11 ∓ 0.12	0.386	0.829
Ferritin (ng/mL)	49.31 ∓ 26.47	60.43 ∓ 27.50	0.322	0.768
Vitamin D (µg/L)	16.55 ∓ 9.35	19.00 ∓ 10.31	0.612	0.884
Folate (ng/mL)	7.27 ∓ 3.20	7.98 ∓ 3.19	0.603	0.884
sT3 (pg/mL)	4.28 ∓ 0.62	4.12 ∓ 0.55	0.549	0.884
sT4 (ng/dL)	1.24 ∓ 0.17	1.29 ∓ 0.18	0.474	0.850
Cortisol (µg/dL)	9.40 ∓ 4.98	10.40 ∓ 4.62	0.634	0.884

The patient group consisted of 103 individuals, including 97 with the AA genotype and 6 with the AG genotype. Exploratory analyses with a small sample size (*n* = 6) are indicated, and results should be interpreted cautiously. Nominal *p*-values were obtained using independent samples *t*-tests. Multiple comparison corrections were applied using the FDR procedure. Adjusted *p*-values < 0.05 were considered statistically significant.

## Data Availability

The original contributions presented in this study are included in the article. Further inquiries can be directed to the corresponding author.
